# Morbidities & outcomes of a neonatal intensive care unit in a complex humanitarian conflict setting, Hajjah Yemen: 2017-2018

**DOI:** 10.1186/s13031-020-00297-7

**Published:** 2020-07-29

**Authors:** Paul Eze, Fatoum Al-Maktari, Ahmed Hamood Alshehari, Lucky Osaheni Lawani

**Affiliations:** 1 Medecins Sans Frontieres OCBA, Barcelona, Spain; 2 Paediatrics Unit, Al Gomhoury Hospital Hajjah City, Hajjah Governorate, Yemen; 3 Department of Paediatrics, Thamar University Faculty of Medicine and Health Sciences, Dhamar, Yemen; 4 Department of Obstetrics & Gynaecology, Alex Ekwueme Federal University Teaching Hospital, Abakaliki, Nigeria

**Keywords:** Preterm neonates, Neonatal mortality, Travel time, Conflict settings, Yemen

## Abstract

**Background:**

The protracted conflict in Yemen has taken a massive toll on the health system, negatively impacting the health of children, especially the most vulnerable age group; the newborns.

**Methods:**

A 2-year retrospective study of admissions into the Neonatal Intensive Care Unit (NICU) in Al-Gomhoury Hospital Hajjah, Northwest Yemen was conducted. Data was analyzed with IBM SPSS® version 25.0 statistical software using descriptive/inferential statistics.

**Results:**

A total of 976 newborns were eligible and included in this study; 506 preterm newborns (51.8%) and 470 term newborns (48.2%). Over half, 549 (56.3%) newborns were admitted within 24 h after birth and 681 (69.8%) newborns travelled for over 60 min to arrive at the NICU. The most common admission diagnoses were complications of prematurity (341; 34.9%), perinatal asphyxia (336; 34.4%), neonatal jaundice (187; 18.8%), and neonatal sepsis (157, 16.1%). The median length of stay in the NICU was 4 days. There were 213 neonatal deaths (Facility neonatal mortality rate was 218 neonatal deaths per 1000 livebirths); 192 (90.1%) were preterm newborns, while 177 (83.1%) were amongst newborns that travelled for more 60 min to reach the NICU. Significant predictors of neonatal deaths are preterm birth (aOR = 3.09, 95% CI: 1.26–7.59, *p* = 0.014 for moderate preterm neonates; aOR = 6.18, 95% CI: 2.12–18.01, *p* = 0.001 for very preterm neonates; and aOR = 44.59, 95% CI: 9.18–216.61, *p* <  0.001 for extreme preterm neonates); low birth weight (aOR = 3.67, 95% CI: 1.16–12.07, *p* = 0.032 for very low birth weight neonates; and aOR = 17.42, 95% CI: 2.97–102.08, *p* = 0.002 for extreme low birth weight neonates); and traveling for more than 60 min to arrive at the NICU (aOR = 2.32, 95% CI: 1.07–5.04, *p* = 0.033). Neonates delivered by Caesarean section had lower odds of death (aOR = 0.38, 95% CI 0.20–0.73, *p* = 0.004) than those delivered by vaginal birth.

**Conclusions:**

Preterm newborns bear disproportionate burden of neonatal morbidity and mortality in this setting which is aggravated by difficulties in accessing early neonatal care. Community-based model of providing basic obstetric and neonatal care could augment existing health system to improve neonatal survival in Yemen.

## Background

About 80% of Yemenis are in need of humanitarian assistance, with about 7.3 million persons severely food insecure, and 3.3 million persons internally displaced. Furthermore, over 8 million people have no access to health care due to the ongoing conflict [1–3]. The current war in Yemen has exacerbated the country’s preexisting challenges including poverty, poor health, and shortage of necessities such as water, fuel, and medications [3–6]. This situation is even worse for women and children. An antenatal coverage of at least one visit of only 60%; about 70% home delivery and less than 45% of births are assisted by skilled attendants; and mortality rate of 148 maternal deaths per 100,000 live births all indicate poor healthcare for women during pregnancy and delivery [7]. As of 2019, Yemen was ranked 177th out of 189 countries on the human development index [8].

Globally, about 2.5 million newborns die each year, accounting for 46% of all deaths in children less than 5 years [9]. In Yemen, the national neonatal mortality rate for the period 2009–2013 was 26 deaths per 1000 livebirths, accounting for 49% of all deaths in children less than 5 years [7]. Indeed, the neonatal period, defined as the first 28 days of life, represents the most vulnerable period in a child’s life [10]. Increasing proportion of the global neonatal mortality; defined as deaths of live born children on or before 28 completed days after birth [11], occur in countries characterized by significant civil conflict and political instability [12]. Conflicts and political instability weaken the governance structure and impairs the infrastructural capacity to provide basic skilled birth attendants, emergency obstetric and newborn care and address neonatal complications leading to an inverse association between political stability and neonatal mortality. Hence, as political instability and humanitarian crisis looms, the neonatal mortality worsens [13–15].

Several studies, however, report difficulty in ascertaining key reproductive, maternal, neonatal and child health (RMNCH) interventions and indicators in conflict settings like Yemen [4, 14, 15], a gap which represents a barrier to improving care for newborns in such settings. This evaluation of morbidity and mortality pattern of newborns was therefore necessary to provide non-existent local data on neonatal health in a complex humanitarian conflict setting.

## Methods

### Study design

This is a 2-year retrospective study of newborns admitted in the Neonatal Intensive Care Unit (NICU) of Al Gomhoury Hospital Hajjah in Hajjah City, Hajjah Governorate, Yemen between 01 January 2017 and 31 December 2018.

### Study setting

**Fig. 1:**
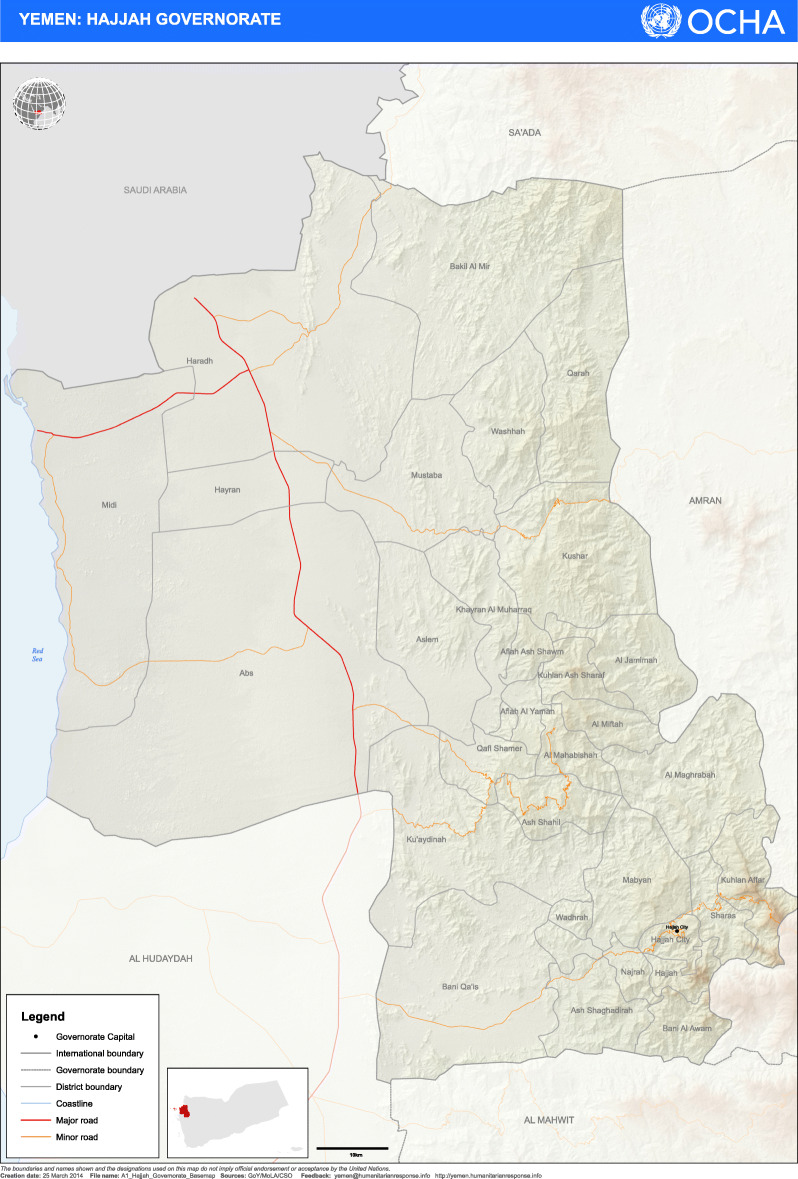
Hajjah Governorate ATTRIBUTION: “Map provided courtesy of the UN Office for the Coordination of Humanitarian Affairs” DISCLAIMER: “The boundaries and names shown and the designations used on this map do not imply official endorsement or acceptance by the United Nations”

Yemen lies in the south of the Arabian Peninsula bordered from the west by Oman and from the north by the Kingdom of Saudi Arabia with the Red Sea to the east and the Indian Ocean to the south. Hajjah governorate is one of the 22 governorates in Yemen, and it is further sub-divided into 31 districts with the political and administrative capital at Hajjah City – Fig. 1. The governorate is mostly mountainous with a low-lying coastal area adjacent to the Red Sea. There are two urban settlements; Hajjah city and Abs. Transportation is mainly by road transport with cars and trucks between districts and motorcycles within Abs and Hajjah City. Movements with donkeys are also common in villages. Civilian air transport was not possible during the period of study due to the conflict.

Hajjah Governorate has about 2.51 million inhabitants; including over 420,000 internally displaced persons [2]. Since there are no camps for internally displaced persons (IDPs), displacement has led to a dispersed population living in makeshift tents [16]. The United Nations (UN) has estimated that over 2 million persons in Hajjah Governorate need humanitarian assistance, with 69% of these in dire acute need [2]. The entire country faces severe food emergency with over 60% of the population at risk [16]. The health care system in Yemen is on the brink of collapse, with over 90% of the population in health needs [2, 3]. The World Health Organization (WHO)‘s Health Resources Availability Monitoring System (HeRAMS) estimates that 49% of the health facilities in Yemen are either not functioning or only partially functioning [17]. There are 6.2 beds per 10,000 of the population in Yemen – less than the 10 beds per bed recommended by WHO [17]. In Hajjah governorate, there are 7.8 health workers (medical doctors, nurses, and midwives) available for 10,000 of the population – less than 22 health workers per 10,000 populations as per United Nations Inter-Agency Standing Committee (IASC)‘s standard [2]. The Governorate battled outbreaks of cholera, diphtheria, dengue fever and measles during the study period [3, 18–21]. Indeed, Hajjah Governorate is one of the worse affected governorates in need of health assistance [2].

Al Gomhoury Hospital Hajjah is a 180-beds hospital in Hajjah City which serves as the referral hospital for Hajjah governorate. The hospital is supported by Non-Governmental Organizations including United Nations Children Fund (UNICEF), World Health Organization (WHO), United Nations Fund for Population Activities (UNFPA) and Medecins Sans Frontieres (MSF). The Saudi Hospital, also in Hajjah City, provides free secondary healthcare. Based on the American Academy of Paediatrics policy statement on the level of neonatal care [22], the Al Gomhoury Hospital Hajjah has a level 3 NICU. Both Saudi Hospital in Hajjah City and Abs General Hospital in Abs (which is supported by MSF) have Level 1 NICU and both hospitals refer newborn babies to Al Gomhoury Hospital Hajjah.

The NICU in Al Gomhoury Hospital has 16 newborn cots and mainly provides care for neonates with medical conditions and admits patients in need of surgical intervention for stabilization prior to referral to Sana’a. The NICU has both in-born and out-born sections and is manned by two neonatologists, one medical doctor and an average of four nursing staff per shift. About 40 newborns are admitted every month. During the period of this study, the NICU had capacity for thermoregulation, intravenous hydration, cup and nasogastric tube feedings, phototherapy, and oxygen support though several oxygen concentrators with nasal cannula. Electrical power was constantly available, thanks to logistics support by donor agencies. Neonates requiring pediatric subspecialty care or surgical care were first stabilized and then referred upwards to Al Sabeen Hospital or Al-Gumhouri Teaching Hospitals in Sana’a, which is about 3 h’ travel by car.

### Data collection

**Fig. 2:**
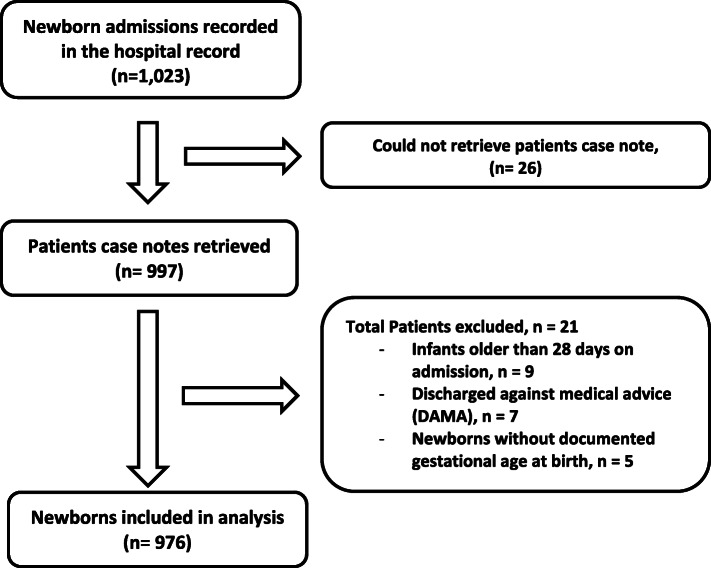
**Flowchart of identification of study population. Flowchart of identification of study population.** Number of total patients admitted by hospital according to hospital records, case notes retrieved, patients excluded, and patients included in the study

Case notes and admission records in the newborn unit were used to extract information on sex, age at admission, residence, gestational age at delivery, weight on admission, place of delivery, diagnoses on admission, main therapies received, duration of admission and discharge outcome. Newborns with missing case notes, infants older than 28 days, and newborns discharged against medical advice (DAMA) were excluded Fig. 2. Data was abstracted by three trained qualified nurses who had diploma and work experience in the hospital. The overall data collection process was supervised by two of the authors.

### Definition and outcomes

Gestational age at birth was the estimated duration (in weeks) of the pregnancy which was determined (in order of preference) by the report on the health card, using the last menstrual period reported by the mother or gestational assessment using the New Ballard’s assessment scale on admission (for newborns up to 7 d postnatal age). When none of these was obtainable, such babies were excluded. Low birthweight was defined as birthweight less than 2500 g (up to and including 2499 g); very low birthweight as birth weight less than 1500 g (up to and including 1499 g); and extremely low birthweight as birthweight less than 1000 g (up to and including 999 g) [23]. Inborn neonates were defined as newborns born in Al Gomhoury Hospital Hajjah while outborn were those born outside the facility (including home delivery). The travel distance from the patients’ homes and the travel time to the NICU were based on the distance (in kilometers) and duration (in minutes) officially provided by the Records and Statistics unit of the Ministry of Public Health and Population (MOPHP) in Hajjah City, Hajjah Governorate. The travel distance and travel time is the distance and time MOPHP staffs required to travel from Hajjah City to the government-owned district health facilities for health mobilization, vaccination, and other health activities. This does not include extra distance and time required to travel on foot from the patients’ home to the usually centrally located district health facility. Also, travel time did not include the added time spent at military check points. Study outcomes were to identify the main neonatal morbidities, the neonatal mortality rate, and key predictors of neonatal mortality.

### Statistical analysis

Data were entered into Microsoft Excel (Microsoft, Redmond, WA, USA), cleaned and transferred to SPSS version 25.0 (IBM, Armonk, NY, USA) for statistical analyses. Normally distributed data were reported using mean while non-normally distributed data were reported using median. Travel distance and travel time were categorized using their median values; 40 Km and 60 min, respectively. Bivariate analyses were performed to determine associations between baseline neonatal characteristics. When both Chi-Square test and Fischer’s Exact Tests were estimated, only the *p*-value of the Fischer’s Exact test is reported. Admission diagnoses were categorized (such as prematurity, perinatal asphyxia, neonatal sepsis, etc.) but a neonate could have multiple documented admission diagnoses. For admission diagnoses and therapies received, reported percentages reflect the number of neonates with the diagnosis and number of children who received the therapy, respectively. Mann–Whitney *U* test was used to estimate the difference in median length of stay (LoS). Multivariate logistic regression analyses were performed to estimate adjusted odds ratios for study outcomes, and to also estimate the effect of travel distance and travel time on the odds of neonatal mortality in the NICU. *P* <  0.05 was used to define statistical significance and all tests were two-tailed.

## Results

A total of 976 newborns (96.4%) were eligible and included for this study (Fig. 2). More than half (549 neonates; 56.3%) of the newborn babies were admitted within 24 h of delivery, and about two in five (372 neonates; 38.1%) were delivered at home. More than half (506 neonates; 51.8%) were delivered preterm (less than 37 completed weeks gestational age). There were 213 neonatal deaths; hence neonatal mortality rate is 218 neonatal deaths per 1000 live births. Weight of the newborn on admission, travel time to the NICU and other demographic and clinical characteristics are shown in Table 1.

**Table 1:** Socio-demographic & clinical characteristics of study neonates in NICU, Hajjah Yemen 2017–2018

Demographic & clinical characteristics	Total Admission (***n*** = 976)	Neonatal mortality (***n*** = 213)	Mortality OR (95% CI)	***P-***value
**Age of newborn**				
− ≤ 24 h	549 (56.3%)	146 (68.5%)	2.45 (1.42–4.22)	< 0.001
− 2 to 7 days	295 (30.2%)	50 (23.5%)	1.38 (0.76–2.50)	0.316
− 8 to 28 days	132 (13.5%)	17 (8.0%)	1.00	
**Sex of newborn**				
− Male	577 (59.1%)	127 (59.6%)	1.03 (0.75–1.40)	0.875
− Female	399 (40.9%)	86 (40.4%)	1.00	
**Residence**				
− Rural	755 (77.4%)	186 (87.3%)	2.35 (1.52–3.63)	< 0.001
− Urban	221 (22.6%)	27 (12.7%)	1.00	
**Place of Delivery**				
− Home delivery	372 (38.1%)	74 (34.7%)	0.83 (0.61–1.14)	0.265
− Health facility	604 (61.9%)	139 (65.3%)	1.00	
**Mode of Delivery**				
− Caesarean section	163 (16.7%)	21 (9.9%)	0.48 (0.29–0.78)	0.003
− Vaginal delivery	813 (83.3%)	192 (90.1%)	1.00	
**Inborn vs Outborn**				
− Outborn	717 (73.5%)	174 (81.7%)	1.81 (1.24–2.65)	0.002
− Inborn	259 (26.5%)	39 (18.3%)	1.00	
**Number of Gestation(s)**				
− Twins & Triplets	83 (8.5%)	25 (11.7%)	1.62 (0.98–2.65)	0.070
− Singleton	893 (91.5%)	188 (88.3%)	1.00	
**Estimated Gestational Age**				
− < 28 weeks	68 (7.0%)	63 (29.6%)	269.40 (98.09–739.92)	< 0.001
− 28 weeks to < 32 weeks	181 (18.5%)	82 (38.5%)	17.71 (10.46–29.98)	< 0.001
− 32 weeks to < 37 weeks	257 (26.3%)	47 (22.1%)	4.79 (2.79–8.21)	< 0.001
− ≥ 37 weeks	470 (48.2%)	21 (9.9%)	1.00	
**Weight on Admission**				
− < 1000 g	55 (5.6%)	51 (24.0%)	243.52 (85.57–696.03)	< 0.001
− 1000 to 1500 g	153 (15.7%)	81 (38.0%)	26.86 (14.85–48.57)	< 0.001
− 1501 to 2499 g	370 (37.9%)	65 (30.5%)	5.09 (2.89–8.97)	< 0.001
− ≥ 2500 g	398 (40.8%)	16 (7.5%)	1.00	
**Travel Distance to the NICU**				
− > 40 Km	475 (48.7%)	141 (66.2%)	2.52 (1.83–3.47)	< 0.001
− ≤ 40 Km	501 (51.3%)	72 (33.8%)	1.00	
**Travel Time to the NICU**				
− > 60 min	681 (69.8%)	177 (83.1%)	2.53 (1.71–3.73)	< 0.001
− ≤ 60 min	295 (30.2%)	36 (16.9%)	1.00	

***Abbreviations*:***OR* Odds Ratio, *CI* Confidence Interval

About a third of the newborns (341 neonates; 34.9%) were admitted for complications of prematurity. However, over two-thirds of the neonatal deaths (157 neonates; 73.7%) were due to complications of prematurity. The major morbidities in this study were prematurity (and its complications), perinatal asphyxia, neonatal jaundice, and neonatal sepsis; but prematurity (and its complications) was the only significant cause of mortality amongst newborns admitted in this study. The overall median length of stay (LoS) in the NICU was 4 d, but 3 d for neonates that died (Mann-Whitney *U* = 67,363.5; *p* <  0.001). Details on the treatments and interventions provided in the NICU are shown in Table 2.

**Table 2:** Admission diagnosis and therapies received by study newborns in NICU, Hajjah Yemen 2017–2018

Clinical Indications	Total Admission (***n*** = 976)	Neonatal mortality (***n*** = 213)	Mortality OR (95% CI)	***P***-value
Admission diagnosis **				
− Prematurity	341 (34.9%)	157 (73.7%)	8.82 (6.23–12.49)	< 0.001
− Perinatal asphyxia	336 (34.4%)	46 (21.6%)	0.45 (0.31–0.64)	< 0.001
− Neonatal jaundice	183 (18.8%)	26 (12.2%)	0.54 (0.34–0.84)	0.007
− Neonatal sepsis	157 (16.1%)	11 (5.2%)	0.23 (0.12–0.43)	< 0.001
− Congenital anomalies	34 (3.5%)	7 (3.2%)	0.93 (0.40–2.16)	1.000
− Others	32 (3.3%)	1 (0.5%)	0.11 (0.02–0.82)	0.015
Therapies received **				
− Antibiotics	965 (98.9%)	212 (99.5%)	2.82 (0.36–22.12)	0.472
− Formal phototherapy	187 (19.2%)	36 (16.9%)	0.82 (0.55–1.23)	0.376
− Oxygen therapy	284 (29.1%)	53 (24.9%)	0.76 (0.54–1.08)	0.147
− Extra feeding support	408 (41.8%)	95 (44.6%)	1.16 (0.85–1.57)	0.388
− Stabilised and referred	47 (4.8%)	N/A	N/A	N/A
**Length of Stay (LoS) in NICU**				
− Median (IQR) in days	4.00 (7.00)	3.00 (6.00)	1.00 (1.00–2.00) ^#^	< 0.001 ^#^

***Abbreviations*:***IQR* Inter-quartile Range, *N/A* Not applicable

** More than one admission diagnosis/therapy could apply

^#^ Difference in median, Confidence interval and p-value for the difference in the median using Mann-Whitney U-test

Sub-analysis of neonatal clinical characteristics based on inborn versus outborn status – Table 3, demonstrates significant differences in neonatal age on admission, mode of delivery and admission diagnosis. Newborn babies born outside the hospital (outborn) have higher odds of presenting after the first day after birth (OR = 1.56, 95% CI: 1.16–2.09, *p* = 0.003); preterm, < 37 completed weeks gestational age (OR = 1.24, 95% CI: 0.93–1.65, *p* = 0.147); delivered by vaginal birth (OR = 18.20, 95% CI: 12.03–27.54, *p* = < 0.001); admitted for complications of prematurity (OR = 1.38, 95% CI: 1.01–1.87, *p* = 0.048); and perinatal asphyxia (OR = 2.02, 95% CI: 1.46–2.79, *p* <  0.001). Outborn neonates have less odds of being admitted for neonatal jaundice (OR = 0.54, 95% CI: 0.39–0.78, *p* = 0.001). Outborn neonates significantly stayed longer on admission than inborn neonates with difference in median LoS = 1.00 day (Mann-Whitney *U* = 95,094.5; *p* = 0.001).

**Table 3:** Sub-analysis of clinical characteristics of neonates born inside (inborn) vs outside (outborn)

Clinical characteristics and outcomes	Inborn (***n*** = 259)	Outborn (***n*** = 717)	OR (95% CI)	***P***-value
**Age on admission**				
− ≤ 24 h	166 (64.1%)	383 (53.4%)	1.56 (1.16–2.09)	0.003
− 2 to 28 days	93 (35.9%)	334 (46.6%)		
**Estimated Gestational Age**				
− < 37 weeks	124 (47.9%)	382 (53.3%)	1.24 (0.93–1.65)	0.147
− ≥ 37 weeks	135 (52.1%)	335 (46.7%)		
**Mode of delivery**				
− Vaginal delivery	132 (51.0%)	681 (95.0%)	18.20 (12.03–27.54)	< 0.001
− Caesarean section	127 (49.0%)	36 (5.0%)		
Admission diagnosis **				
− Prematurity	77 (29.7%)	264 (36.8%)	1.38 (1.01–1.87)	0.048
− Perinatal asphyxia	61 (23.6%)	275 (38.4%)	2.02 (1.46–2.79)	< 0.001
− Neonatal jaundice	67 (25.9%)	116 (16.2%)	0.55 (0.39–0.78)	0.001
− Neonatal sepsis	48 (18.5%)	109 (15.2%)	0.79 (0.54–1.15)	0.236
− Congenital anomalies	5 (1.9%)	29 (4.1%)	2.14 (0.82–5.59)	0.119
− Others	23 (8.9%)	9 (1.3%)	0.13 (0.06–0.29)	< 0.001
**Length of Stay (LoS) in NICU**				
− Median (IQR) in days	3.00 (6.50)	4.00 (8.00)	1.00 (0.00–2.00) ^#^	0.001 ^#^

**Abbreviations:** OR, Odds Ratio. CI, Confidence Interval

** More than one admission diagnosis could apply

^#^ Difference in median, Confidence interval and p-value for the difference in the median using Mann-Whitney U-test

Table 4 shows the results of multivariate logistics regression analysis for the key predictors of neonatal mortality. After adjusting for the admission diagnosis, and intervention/therapies received identified from univariate analysis as significant co-variates, statistically significant key predictors are preterm birth (aOR = 3.09, 95% CI: 1.26–7.59, *p* = 0.014 for moderate preterm neonates; aOR = 6.18, 95% CI: 2.12–18.01, *p* = 0.001 for very preterm neonates; and aOR = 44.59, 95% CI: 9.18–216.61, *p* <  0.001 for extreme preterm neonates); low birth weight (aOR = 3.67, 95% CI: 1.16–12.07, *p* = 0.032 for very low birth weight neonates; and aOR = 17.42, 95% CI: 2.97–102.08, *p* = 0.002 for extreme low birth weight neonates); and traveling for more than 60 min to arrive at the NICU (aOR = 2.32, *p* = 0.033). Neonates delivered by Caesarean section had lower odds of death (aOR = 0.38, 95% CI: 0.20–0.73, *p* = 0.004) than those delivered by vaginal birth.

**Table 4:** Multivariate logistics regression analysis for socio-demographic predictors of facility neonatal mortality in the NICU

Demographic & clinical Variables	Reference	Adjusted OR	95% CI	***P-***value
**Newborn age at admission**				
− ≤ 24 h	8 to 28 days	2.51	1.22–5.13	0.012
− 2 to 7 days	8 to 28 days	1.46	0.69–3.08	0.323
**Sex of newborn**				
− Male	Female	1.14	0.75–1.73	0.546
**Residence**				
− Rural	Urban	1.85	0.94–3.66	0.077
**Place of delivery**				
− Home	Health facility	0.93	0.40–1.98	0.098
**Mode of delivery**				
− Caesarean section	Vaginal	0.38	0.20–0.73	0.004
**Estimated gestational age at birth**				
− 32–37 weeks	≥ 37 weeks	3.09	1.26–7.59	0.014
− 28–32 weeks	≥ 37 weeks	6.18	2.12–18.01	0.001
− < 28 weeks	≥ 37 weeks	44.59	9.18–216.61	< 0.001
**Admission weight**				
− 1501 to 2499 g	≥ 2500 g	1.65	0.64–4.28	0.303
− 1000 to 1500 g	≥ 2500 g	3.67	1.16–12.07	0.032
− < 1000 g	≥ 2500 g	17.42	2.97–102.08	0.002
**Travel distance**				
− > 40 Km	≤ 40 Km	1.44	0.83–2.47	0.192
**Travel time**				
− > 60 min	≤ 60 min	2.32	1.07–5.04	0.033

***Abbreviations***: *OR* Odds Ration, *CI* Confidence Interval

^**#**^ Adjusted for sex of newborn, age of newborn at admission, estimated gestational age at birth, admission weight, place of residence, place of delivery, mode of delivery, number of gestation (singleton or multiple), travel time and admission diagnoses

## Discussions

Overall, this study showed that the main neonatal morbidities in this context are prematurity and its complications, perinatal asphyxia, neonatal jaundice, and neonatal sepsis. Prematurity complications and perinatal asphyxia account for about a third of NICU admissions each; and are the most predominant cause of newborn morbidity. Furthermore, similar studies conducted in Sana’a before the current conflict also demonstrated prematurity and its complications as the most predominant cause of NICU admissions [24]. However, the proportion reported in our study is significantly higher suggesting an increasing influence of the factors that precipitate preterm delivery in Yemen. Also, high prevalence of perinatal asphyxia and neonatal sepsis in our study is indicative of the uncharacteristic settings deliveries are conducted in Yemen, as about 70% of births occurred at home and less than 45% of births were attended by a skilled attendant [7].

Facility neonatal mortality rate reported in this study was 218 neonatal deaths per 1000 live births which is comparatively lower than the 232 neonatal deaths per 1000 live births reported by a similar study in Al-Gumhouri Hospital in Sana’a Yemen prior to the onset of this conflict [24]. Although these rates do not represent the totality of the newborn in Yemen, as they only represent deaths amongst newborns at the health facility, they however offer an important glimpse on the state of health of newborns in Yemen. Even So, these rates are substantially higher than the neonatal mortality rates for NICU admissions reported in other settings [25–28]. Our study also showed that neonates born by Caesarean section had 52% less odds of death than those born with normal vaginal birth as they were more likely birthed in a hospital where they received closer monitoring from more specialized health workers (medical doctor, nurses and midwives) than those born by vaginal birth; who mainly delivered at home under unsterile conditions with delivery conducted by unskilled birth attendant [7, 29].

Our study shows that amongst other preventable factors responsible for the high neonatal mortality rate in this setting, geographical accessibility to healthcare remains a challenge. More than two-thirds of the newborns travelled for over an hour to access healthcare which further delays access to healthcare and is counterproductive to efforts to improve neonatal health in this setting. While it’s obvious that Yemen is in urgent need of huge human and infrastructural investments in hospitals, clinics and maternity homes to reverse the dismal health indices made worse by the ongoing conflict [1, 2, 6, 17], our results suggest that geographical barriers in accessing emergency obstetric and neonatal services will still be a challenge. A decentralized model of providing basic obstetric and neonatal care could potentially circumvent these barriers. Home delivery remains common in Yemen despite obvious risks to maternal and newborn health [7, 29]. A programme to train and supervise community midwives (CMW) and traditional birth attendants (TBA), and provide them with appropriate supplies, medicines and equipment could extend basic maternal and neonatal care to women’ homes and improve referral to hospitals for specialist care [30, 31]. Pragmatically linking the CMWs and TBAs in this programme with traditional authorities in the community will enhance community ownership, ensure regular reporting and bolster overall health outcomes. There is need, therefore, for further studies, preferably qualitative, to better understand the issues influencing mothers’ choice to birth at home and how these can be tackled to improve neonatal health in these settings.

Some limitations of this study include being a hospital-based study which fails to capture neonatal deaths in the community. The study also counted multiple diagnosis reported in the case notes for each patient which could inadvertently lead to overestimation of some disease conditions. Additionally, the study did not utilize available technologies to determine distance travelled and time travelled by the patients, which might have introduced some bias and potential overestimation of these variables. However, access restrictions by the authorities and safety concerns could not make us of GPS technologies possible. Yet, it is important that future studies utilize these technologies to eliminate these biases. Many cases present to the NICU without their mothers or the mother were unaware of the gestational age at birth. This study thus relied on the gestational age assessment using the New Ballard’s assessment. Finally, inability to have postmortem assessment to determine the pathologic cause of death could have led to overestimation of deaths due to prematurity.

## Conclusions

Preterm newborns bear a disproportionate burden of neonatal morbidity and mortality in this context which is worsened by geographical difficulties in accessing early neonatal care. A community-based decentralized model of providing basic obstetric and neonatal care could potentially circumvent these geographical barriers, augment existing neonatal healthcare system and improve neonatal survival in Yemen.

## Data Availability

The data that support the findings of this study are available from the Al Gomhoury Hospital Hajjah in Hajjah City, Hajjah Governorate, Yemen; but restrictions apply to the availability of these data, which were used under permission for the current study, and so are not publicly available. Data are, however, available from the authors upon reasonable request and with permission of Al Gomhoury Hospital Hajjah, Hajjah City, Hajjah Governorate, Yemen.

## Abbreviations

NICU: Neonatal Intensive Care Unit
CS: Caesarean section
DAMA: Discharge Against Medical Advice
MOPHP: Ministry of Public Health and Population
CMW: Community Midwife
TBA: Traditional Birth Attendants
IDP: Internally displaced persons
